# Autonomous Landing Strategy for Micro-UAV with Mirrored Field-of-View Expansion

**DOI:** 10.3390/s24216889

**Published:** 2024-10-27

**Authors:** Xiaoqi Cheng, Xinfeng Liang, Xiaosong Li, Zhimin Liu, Haishu Tan

**Affiliations:** 1 School of Mechatronic Engineering and Automation, Foshan University, Foshan 528225, China; chexqi@163.com (X.C.); 13622777987@163.com (Z.L.); 2 Guangdong Provincial Key Laboratory of Industrial Intelligent Inspection Technology, Foshan University, Foshan 528225, China; liangxinfeng2023@163.com (X.L.); lixiaosong@buaa.edu.cn (X.L.); 3 School of Physics and Optoelectronic Engineering, Foshan University, Foshan 528225, China

**Keywords:** micro unmanned aerial vehicle, autonomous landing, camera-IMU extrinsic calibration, angle-of-view conversion, field-of-view expansion

## Abstract

Positioning and autonomous landing are key technologies for implementing autonomous flight missions across various fields in unmanned aerial vehicle (UAV) systems. This research proposes a visual positioning method based on mirrored field-of-view expansion, providing a visual-based autonomous landing strategy for quadrotor micro-UAVs (MAVs). The forward-facing camera of the MAV obtains a top view through a view transformation lens while retaining the original forward view. Subsequently, the MAV camera captures the ground landing markers in real-time, and the pose of the MAV camera relative to the landing marker is obtained through a virtual-real image conversion technique and the R-PnP pose estimation algorithm. Then, using a camera-IMU external parameter calibration method, the pose transformation relationship between the UAV camera and the MAV body IMU is determined, thereby obtaining the position of the landing marker’s center point relative to the MAV’s body coordinate system. Finally, the ground station sends guidance commands to the UAV based on the position information to execute the autonomous landing task. The indoor and outdoor landing experiments with the DJI Tello MAV demonstrate that the proposed forward-facing camera mirrored field-of-view expansion method and landing marker detection and guidance algorithm successfully enable autonomous landing with an average accuracy of 0.06 m. The results show that this strategy meets the high-precision landing requirements of MAVs.

## 1. Introduction

In the past decade, the flight performance and endurance of unmanned aerial vehicles (UAVs) have significantly advanced, playing a major role in both military and civilian fields. Micro-UAVs (MAVs), due to their small size, low cost, high speed, and strong maneuverability, have been widely used in autonomous flight mission areas such as metal corrosion detection [1], underground rescue [2], pipeline inspection [3], and rubble surveying [4].

Positioning and landing technologies are critical for the autonomous flight missions of UAVs. During the landing phase, accurately identifying the landing site and achieving a precise touchdown without being affected by external environmental interference presents a significant challenge. Traditional GPS-based positioning strategies often fall short of meeting the high-precision landing requirements of UAVs. As a result, many studies have introduced new sensor-assisted approaches for UAV landing control, including ultra-wideband beacons [5], infrared cameras [6], ultrasonic sensors [7], radar stations [8], and LiDAR sensors [9]. In recent years, with the advancement of computer vision technology, vision-based autonomous landing for UAVs has developed rapidly. Technologies such as wide-field-of-view and depth imaging [10], infrared beacon detection [11], and low-light image enhancement for nighttime conditions [12] have significantly improved the robustness of UAV landing performance. Vision-based positioning technology, which does not rely on additional sensors, offers strong anti-interference capability and high accuracy [13,14]. At close range, vision sensors can determine the relative pose between the UAV and the landing marker with sub-millimeter precision [15], which is crucial for achieving accurate landings.

For quadrotor MAVs to maintain lightweight construction, they are typically equipped with only a low-resolution monocular camera. Building on this, Wendel et al. [16] proposed a fuzzy visual servoing method using a forward-facing camera to acquire pose information; Yang et al. [17] introduced an onboard monocular vision system for autonomous takeoff, hovering, and landing of MAVs. The forward-facing camera allows the MAV to detect the landing area before descent, but due to its fixed viewpoint and limited field-of-view, the MAV finds it challenging to complete the entire autonomous landing process solely through visual positioning techniques. To address this issue, previous research has explored using active gimbal systems [18] to control the camera angle, and some studies have implemented dual-camera systems [19] to expand the MAVs’ field-of-view, enabling visual SLAM and improved positioning. However, the limited payload capacity of MAVs makes it difficult to maintain stable flight when equipped with gimbals or dual-camera systems, negatively impacting landing accuracy. In response, Zhao et al. [20] embedded a lightweight IMU module into the MAV, providing auxiliary positioning when the camera loses track of the landing marker. However, this approach did not achieve optimal landing speed.

Other studies have shifted towards using visual tracking systems in the landing area to capture and position the MAV. For example, Zou et al. [21] installed dual-color LEDs on the bottom of a DJI Tello MAV, and Lee et al. [22] attached paper markers, with ground-based visual modules detecting these LEDs or markers to provide landing instructions to the MAV. Nguyen et al. [23] proposed installing a panoramic camera in the landing area and using the YOLOv7 algorithm to identify the MAV’s position. While these methods reduce the MAV’s payload, they require prior setup of landing area equipment, limiting their applicability in different scenarios. To enhance the flexibility of vision-based landing tasks, Eberli et al. [24] used a forward-facing camera to detect the landing marker and estimate distance, switching to a downward-facing micro-monocular camera as the MAV approached the marker. Although this method enables dual-perspective observation, processing the images from two cameras increases the system’s complexity. Mu et al. [25] proposed a camera angle-of-view conversion method where a lens angled at 45° to the horizontal plane is mounted in front of the original forward-facing camera on a DJI Tello MAV, converting the forward view into a top view. The YOLOv5 algorithm was then used to detect the landing marker in real-time. While this angle-of-view conversion strategy provided a convenient top view for landing, it sacrificed the advantage of long-range positioning offered by the forward view.

**Figure 1:**
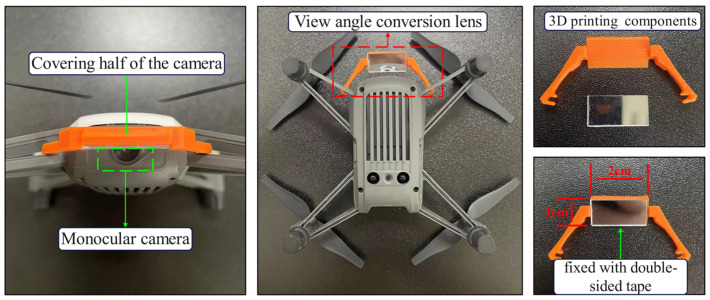
MAV and onboard camera equipped with a mirrored field-of-view expansion lens.

To address these challenges, this study proposes a novel mirrored field-of-view expansion system for the forward-facing camera of MAVs. As shown in Figure 1, an angle- of-view conversion lens is installed on the MAV forward-facing camera, converting the upper half of the camera’s view to a top view while retaining the original forward view in the lower half. This approach expands the field-of-view while maintaining the lightweight design of the MAV. The contributions of this work are as follows:

1. We introduce a mirrored field-of-view expansion system that solves the problem of landing difficulties caused by limited forward-facing camera views during autonomous landing.
2. We design a coarse-to-fine pose estimation algorithm based on virtual-real image transformation, enhancing the recognition performance of landing markers.

The remainder of this paper is organized as follows: Section 2 introduces the proposed mirrored field-of-view expansion system. Section 3 presents the vision-based autonomous landing method for MAVs. Section 4 discusses the indoor and outdoor landing experiments conducted with the DJI Tello MAV and analyzes the experimental results. Finally, Section 5 provides the conclusions.

## 2. Mirrored Field-of-View Expansion System

To address the limitation of the narrow field-of-view in MAV cameras, it is necessary to upgrade the system by equipping additional hardware. We propose a field-of-view expansion solution that includes both hardware and algorithms, making it easier for other researchers to apply this method to their own MAV systems.

The onboard camera of the DJI Tello MAV can only capture a forward-facing view, and the original body weight is just 80 g. To accomplish vision-based landing tasks, it is necessary to improve the narrow field-of-view of the MAV while ensuring it is not burdened by excessive weight. A lens weighing only 5 g was installed in front of the forward-facing camera, angled at 30° from the horizontal plane, and securely attached to the MAV’s arm. As shown in Figure 1, this lens consists of an optical element and a 3D-printed support, covering half of the MAV camera’s field-of-view.

**Figure 2:**
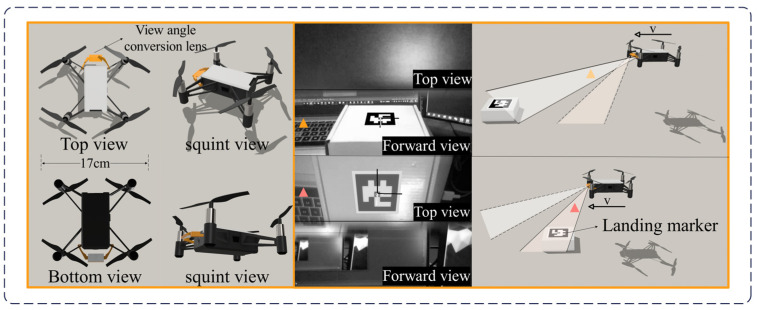
Camera views after installing the lens.

With this lens, the top view can be reflected onto the upper part of the camera’s view, while the forward view remains in the lower part of the camera’s view. Figure 2 shows images captured during the flight of the DJI Tello MAV after the lens was installed. It can be observed that when the MAV is farther from the target, the target can be captured through the forward view. As the MAV approaches the target, the target gradually transitions from the forward view to the top view, allowing it to be captured through the top view.

Therefore, this camera field-of-view expansion system enables the MAV’s forward-facing camera single-view perspective to be extended into a dual-view perspective, incorporating both forward and top views. This allows for the calculation of the pose relationship between the camera and the ArUco markers in the landing area by capturing these markers.

## 3. Vision-Based Autonomous Landing Method for MAV

**Figure 3:**
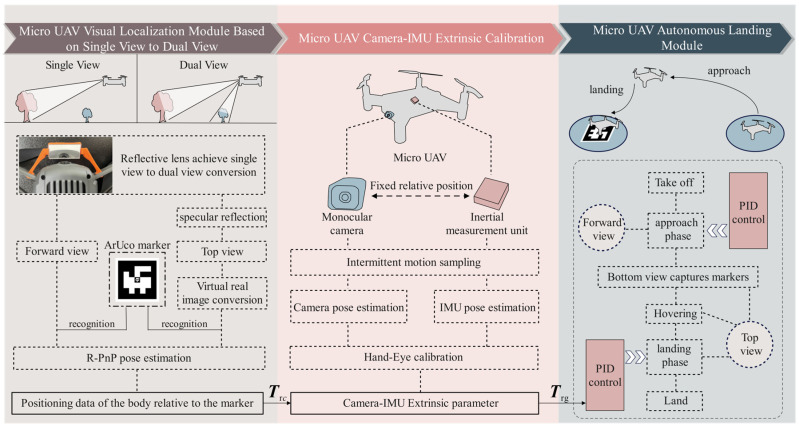
Process of the vision-based autonomous landing method for MAV.

The vision-based MAV autonomous landing framework, as shown in Figure 3, consists of the MAV visual positioning module based on the mirrored field-of-view expansion system, the camera-IMU extrinsic calibration module, and the MAV autonomous landing strategy module.

In the MAV visual positioning module based on the mirrored field-of-view expansion system, a coarse-to-fine MAV pose estimation algorithm is designed in Section 3.1. When the MAV is far from the landing marker, the visual preprocessing module extracts the outer contour of the marker to obtain the corner coordinates. As the MAV becomes closer to the target, the corner coordinates are extracted using OpenCV’s built-in ArUco marker detection algorithm. The pose of the MAV camera relative to the landing marker is then calculated using the robust Perspective-n-Point (R-PnP) algorithm [26]. Additionally, in Section 3.2, a virtual-real image conversion model based on a planar mirror is proposed to address the pose estimation problem in the top view’s mirror imaging, enabling dual-view pose estimation for the system.

The camera-IMU extrinsic calibration module employs a camera-IMU extrinsic calibration technique based on intermittent motion sampling. By simultaneously capturing images of the calibration board with the MAV camera and collecting the attitude data from the IMU, the hand-eye calibration algorithm is used to calculate the pose transformation between the camera and the IMU. This transformation allows the pose relationship between the camera and the landing marker to be converted into a pose relationship between the IMU and the landing marker. Since the IMU coordinate axes are aligned with the MAV’s body center coordinate axes, the pose relationship between the IMU and the landing marker is also regarded as the pose relationship between the MAV body and the landing marker.

The MAV autonomous landing strategy module sequentially executes the four flight phases: takeoff, approach, descent, and landing. During the approach and descent phases, the MAV acquires positioning information and uses a PID algorithm for flight control.

### 3.1. Coarse-to-Fine MAV Pose Estimation Algorithm

**Figure 4:**
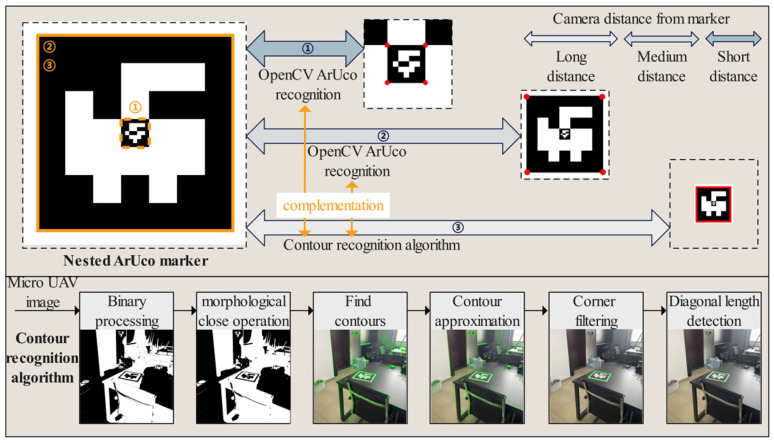
Coarse-to-fine landing marker recognition.

Considering that MAVs are often equipped with low-resolution cameras and have limited bandwidth and transmission rates with the host, the recognizability of landing markers needs to be fully considered when using vision for positioning. ArUco markers are binary matrices composed of black and white grids that can generate patterns with different IDs, maintaining ease of recognition characteristics while also carrying ID information. As shown in Figure 4, this paper designs a nested ArUco marker, consisting of two ArUco markers of different sizes arranged in a 5 × 5 grid. ArUco detection is supported by the OpenCV open-source library. For an ArUco marker with a side length of 25 cm, when the marker plane is placed perpendicular to the camera’s optical axis, the maximum horizontal distance at which it can be recognized is 9 ± 1 m, with specific variations depending on lighting conditions. To increase the recognition distance, we propose a new marker contour extraction method that extends the maximum horizontal recognition distance of the ArUco marker to 11 ± 1 m.

Our proposed ArUco marker contour extraction method consists of five steps. First, when the host receives the image, it performs binarization. Next, a morphological closing operation is applied to the binarized image to remove small black areas within the foreground objects while preserving their size. This step helps prevent the ArUco marker from becoming connected with other regions during the subsequent contour detection. Then, the OpenCV contour detection algorithm is used to extract the contours of the white areas. The contours are then approximated to simplify their shapes while retaining their main features. Finally, the contours are filtered to select those with four corners and roughly equal diagonal lengths, identifying them as the contours of the ArUco marker.

As shown in Figure 4, this method divides the ArUco marker recognition process into three stages: far, medium, and near. When the MAV is far from the ArUco marker (The distance is more than 30 times the size of the external target), the recognition rate of the ArUco marker by the OpenCV algorithm is relatively low. During this stage, the primary approach is to use the marker contour extraction method to extract the outer contour of the marker and obtain its four corner points. When the MAV is at a moderate distance from the ArUco marker (The distance is 5–30 times the size of the external marker), the recognition rate of the ArUco marker by the OpenCV algorithm increases. The contour extraction algorithm is used during gaps where the OpenCV algorithm fails to recognize the marker, thereby improving the overall recognition success rate. When the MAV is close to the ArUco marker (The distance is less than 5 times the size of the external marker), the OpenCV algorithm will recognize the inner ArUco marker. In actual flight, the switching between the three recognition stages is not simply set according to the range of distance, but a detection priority is set. The recognition priority of the external marker is higher than that of the internal marker, and the priority of the OpenCV algorithm is higher than that of the method of extracting the outline of the marker. Therefore, there is no computational redundancy caused by the simultaneous recognition of external and internal markers, which enables smooth switching between stages.

In summary, this contour extraction algorithm not only enhances the ability to recognize ArUco markers at long distances but also complements the OpenCV ArUco recognition algorithm, thereby improving the frame rate for marker recognition across far, medium, and near distances.

After recognizing the ArUco marker, the corner points of the marker are extracted. Given the three-dimensional coordinates of the marker reference points and their corresponding two-dimensional projected pixel coordinates, solving for the camera’s external parameters is commonly referred to as the Perspective-n-Point (PnP) problem. Among the various PnP solving methods, the R-PnP algorithm used in this paper is a fast and robust solution. This method is particularly effective when the number of 2D-3D point pairs is limited, as in the case of the ArUco markers used in this study, which have only four sets of 2D-3D point pairs.

### 3.2. Virtual-Real Image Conversion Model for Mirror Reflection

**Figure 5:**
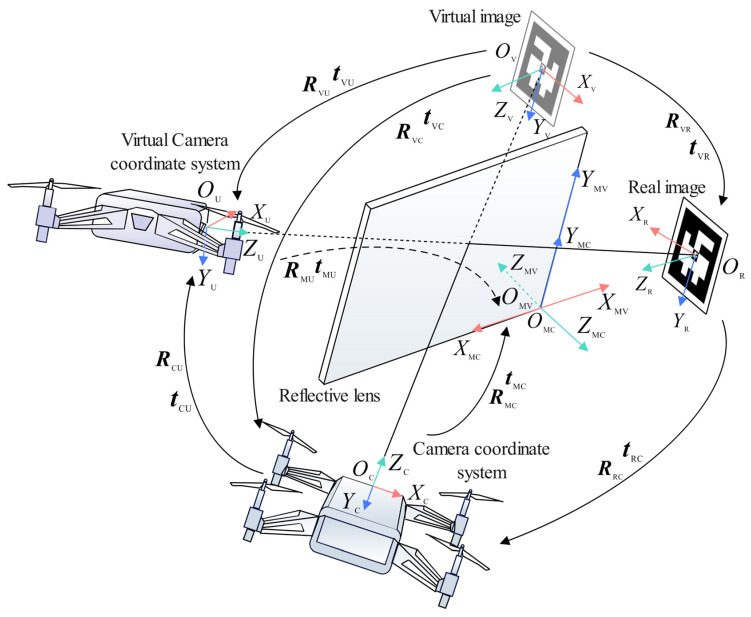
Virtual-real image conversion model based on mirror reflection.

In the visual landing strategy described in this paper, the position of the midpoint of the ArUco landing marker within the MAV’s body coordinate system is used to guide the MAV’s autonomous landing. Therefore, it is first necessary to derive the pose transformation relationship between the MAV camera and the landing marker. As shown in Figure 5, the real landing marker is reflected through the lens into the camera, and the landing marker observed in the camera’s view is the virtual image of the real landing marker. After the corner coordinates of the landing marker are recognized, the R-PnP algorithm can be used to solve the pose transformation between the virtual image of the landing marker and the MAV camera. Using the virtual-real image conversion model proposed below, the pose transformation relationship between the real camera and the virtual camera can be obtained, ultimately yielding the pose transformation relationship between the real landing marker and the MAV camera.

When the top view of the MAV camera captures the landing marker, as shown in Figure 5, the coordinate system of the real camera is defined as $O_{C}X_{C}Y_{C}Z_{C}$, the coordinate system of the virtual camera is defined as $O_{U}X_{U}Y_{U}Z_{U}$, the coordinate system at the center of the real landing marker is defined as $O_{R}X_{R}Y_{R}Z_{R}$, and the coordinate system at the center of the landing marker’s virtual image is defined as $O_{V}X_{V}Y_{V}Z_{V}$. On the front side of the lens, a coordinate system $O_{\mathrm{MC}}X_{\mathrm{MC}}Y_{\mathrm{MC}}Z_{\mathrm{MC}}$ is established relative to the real camera, and on the back side of the lens, a coordinate system $O_{\mathrm{MV}}X_{\mathrm{MV}}Y_{\mathrm{MV}}Z_{\mathrm{MV}}$ is established relative to the virtual camera.

It is known that coordinate systems $O_{\mathrm{MC}}X_{\mathrm{MC}}Y_{\mathrm{MC}}Z_{\mathrm{MC}}$ and $O_{\mathrm{MV}}X_{\mathrm{MV}}Y_{\mathrm{MV}}Z_{\mathrm{MV}}$ are related by a 180° rotation about axis $Y_{\mathrm{MC}}$, so the relationship between $O_{\mathrm{MC}}X_{\mathrm{MC}}Y_{\mathrm{MC}}Z_{\mathrm{MC}}$ and $O_{\mathrm{MV}}X_{\mathrm{MV}}Y_{\mathrm{MV}}Z_{\mathrm{MV}}$ can be expressed as:

(1) $$\left[\begin{array}{c} X_{\mathrm{MV}}\\ Y_{\mathrm{MV}}\\ Z_{\mathrm{MV}} \end{array}\right]=R_{0}\cdot \left[\begin{array}{c} X_{\mathrm{MC}}\\ Y_{\mathrm{MC}}\\ Z_{\mathrm{MC}} \end{array}\right]$$

where $R_{0}=\left[\begin{array}{ccc} -1 & 0 & 0\\ 0 & 1 & 0\\ 0 & 0 & -1 \end{array}\right]$.

**Figure 6:**
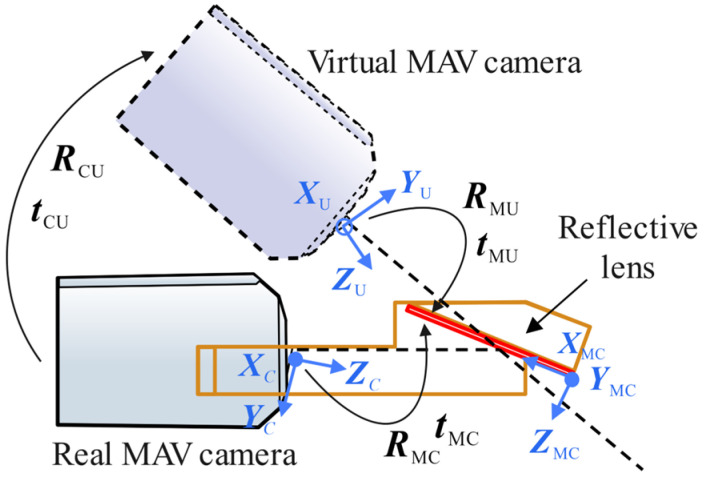
Cross-sectional view of the mirrored field-of-view expansion lens.

As shown in Figure 6, the installation angle and position of the planar mirror relative to the MAV camera are known, allowing the calculation of the rotation $R_{\mathrm{MC}}$ and translation $t_{\mathrm{MC}}$ from the camera coordinate system $O_{C}X_{C}Y_{C}Z_{C}$ to the front side of the planar mirror coordinate system $O_{\mathrm{MC}}X_{\mathrm{MC}}Y_{\mathrm{MC}}Z_{\mathrm{MC}}$. The equations are as follows:

(2) $$R_{\mathrm{MC}}=\left[\begin{array}{ccc} r_{11} & r_{12} & r_{13}\\ r_{21} & r_{22} & r_{23}\\ r_{31} & r_{32} & r_{33} \end{array}\right],t_{\mathrm{MC}}=\left[\begin{array}{c} t_{1}\\ t_{2}\\ t_{3} \end{array}\right]$$

Set the rotation angles of the real camera around the coordinate axes $X_{\mathrm{MC}}$, $Y_{\mathrm{MC}}$ and $Z_{\mathrm{MC}}$ be $\alpha_{\mathrm{MC}}$, $\beta_{\mathrm{MC}}$ and $\omega_{\mathrm{MC}}$, respectively, and the rotation angles of the virtual camera around the coordinate axes $X_{\mathrm{MV}}$, $Y_{\mathrm{MV}}$ and $Z_{\mathrm{MV}}$ be $\alpha_{\mathrm{MU}}$, $\beta_{\mathrm{MU}}$ and $\omega_{\mathrm{MU}}$, respectively. The translation amounts of the real camera relative to the front coordinate axes $X_{\mathrm{MC}}$, $Y_{\mathrm{MC}}$ and $Z_{\mathrm{MC}}$ of the planar mirror are $x_{\mathrm{MC}}$, $y_{\mathrm{MC}}$ and $z_{\mathrm{MC}}$, respectively, and the translation amounts of the virtual camera relative to the back coordinate axes $X_{\mathrm{MV}}$, $Y_{\mathrm{MV}}$ and $Z_{\mathrm{MV}}$ of the planar mirror are $x_{\mathrm{MU}}$, $y_{\mathrm{MU}}$ and $z_{\mathrm{MU}}$, respectively. Thus, we have:

(3) $$\left\{\begin{array}{l} \alpha_{\mathrm{MC}}=\alpha_{\mathrm{MU}}\\ \beta_{\mathrm{MC}}=-\beta_{\mathrm{MU}}\\ \omega_{\mathrm{MC}}=-\omega_{\mathrm{MU}} \end{array}\right.,\left\{\begin{array}{l} x_{\mathrm{MC}}=-x_{\mathrm{MU}}\\ y_{\mathrm{MC}}=y_{\mathrm{MU}}\\ z_{\mathrm{MC}}=z_{\mathrm{MU}} \end{array}\right.$$

Substituting Equation (3) into Equation (2), the pose transformation from the virtual camera to the back side of the planar mirror can be obtained as:

(4) $$R_{\mathrm{MU}}=\left[\begin{array}{ccc} r_{11} & -r_{12} & -r_{13}\\ -r_{21} & r_{22} & r_{23}\\ -r_{31} & r_{32} & r_{33} \end{array}\right],t_{\mathrm{MU}}=\left[\begin{array}{c} -t_{1}\\ t_{2}\\ t_{3} \end{array}\right]$$

From the rotational relationship between the front and back sides of the planar mirror in Equation (1), and the rotational matrix $R_{\mathrm{MU}}$ and translation vector $t_{\mathrm{MU}}$ from the virtual camera to the back side of the planar mirror in Equation (4), the rotational matrix $R_{M}^{U}$ and translation vector $t_{M}^{U}$ from the virtual camera to the front side of the planar mirror can be derived as:

(5) $$\left\{\begin{array}{l} R_{M}^{U}=R_{\mathrm{MU}}\cdot R_{0}\\ t_{M}^{U}=t_{\mathrm{MU}} \end{array}\right.$$

Both the real camera and the virtual camera are established in the front-side coordinate system $O_{\mathrm{MC}}X_{\mathrm{MC}}Y_{\mathrm{MC}}Z_{\mathrm{MC}}$ of the planar mirror. Based on Equations (2) and (5), the pose relationship between the real camera and the virtual camera can be derived as:

(6) $$\left\{\begin{array}{l} R_{\mathrm{CU}}=R_{M}^{U}\cdot (R_{\mathrm{MC}}{)}^{-1}\\ t_{\mathrm{CU}}=t_{M}^{U}+R_{\mathrm{CU}}\cdot (-t_{\mathrm{MC}}) \end{array}\right.$$

Equation (6) can be further simplified as:

(7) $$\left\{\begin{array}{l} R_{\mathrm{CU}}=R_{\mathrm{MU}}\cdot R_{0}\cdot (R_{\mathrm{MC}}{)}^{-1}\\ t_{\mathrm{CU}}=t_{M}^{U}-R_{\mathrm{MU}}\cdot R_{0}\cdot (R_{\mathrm{MC}}{)}^{-1}\cdot t_{\mathrm{MC}} \end{array}\right.$$

By combining the pose transformation $\left(R_{\mathrm{VC}},t_{\mathrm{VC}}\right)$ from the landing marker’s virtual image to the MAV camera, the pose relationship between the landing marker’s virtual image and the virtual camera can be obtained as:

(8) $$\left\{\begin{array}{l} R_{\mathrm{VU}}=R_{\mathrm{CU}}\cdot R_{\mathrm{VC}}\\ t_{\mathrm{VU}}=t_{\mathrm{CU}}+R_{\mathrm{VU}}\cdot t_{\mathrm{VC}} \end{array}\right.$$

Referring to Equations (2) and (4), $R_{\mathrm{VU}}$ and $t_{\mathrm{VU}}$ are defined as:

(9) $$R_{\mathrm{VU}}=\left[\begin{array}{ccc} g_{11} & g_{12} & g_{13}\\ g_{21} & g_{22} & g_{23}\\ g_{31} & g_{32} & g_{33} \end{array}\right],t_{\mathrm{VU}}=\left[\begin{array}{c} k_{1}\\ k_{2}\\ k_{3} \end{array}\right]$$

The pose relationship between the real landing marker and the real camera is then:

(10) $$R_{\mathrm{RC}}=\left[\begin{array}{ccc} g_{11} & -g_{12} & -g_{13}\\ -g_{21} & g_{22} & g_{23}\\ -g_{31} & g_{32} & g_{33} \end{array}\right],t_{\mathrm{RC}}=\left[\begin{array}{c} -k_{1}\\ k_{2}\\ k_{3} \end{array}\right]$$

### 3.3. Camera-IMU Extrinsic Calibration Method Based on MAV

**Figure 7:**
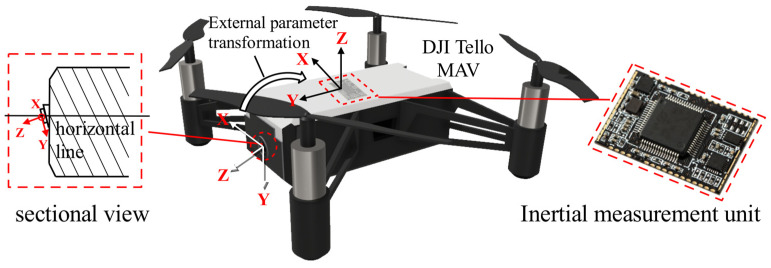
The extrinsic parameters between the camera and IMU.

In the previous section, the pose transformation from the landing marker to the MAV camera was solved, allowing us to obtain the spatial position of the landing marker relative to the camera’s coordinate system. However, relying solely on this position information for landing localization is challenging. Therefore, we need to transform the coordinate system, converting the position of the landing marker from the camera’s coordinate system to the body coordinate system. To achieve this, we must determine the extrinsic pose transformation relationship between the camera and the body. This transformation cannot be directly estimated by eye, as the forward-facing camera of the MAV, such as the DJI Tello used in this study, does not have its optical axis perpendicular to the body. Instead, the camera is slightly tilted downward, as shown in Figure 7. Hence, a more accurate method is required to estimate the camera-to-body extrinsic parameters. Since the IMU’s coordinate system is typically aligned with the UAV body, with the origin representing the center of the MAV body, we extend previous research methods by employing camera-to-IMU extrinsic calibration techniques [27] to solve the MAV camera-to-body extrinsic estimation problem.

**Figure 8:**
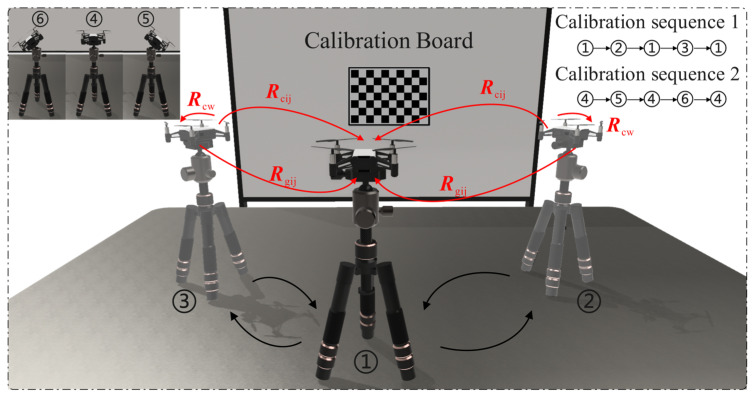
Procedure for calibrating the extrinsic parameters between the camera and IMU.

When calibrating the extrinsic parameters between the camera and IMU, the MAV needs to be fixed on a tripod and placed in front of a checkerboard calibration board. Both the camera and IMU are operated simultaneously, and data are collected by following the motion trajectory 1-2-1-3-1, as shown in Figure 8, with the camera’s optical axis always facing the calibration board. To introduce rotation in multiple directions, data are further collected following the trajectory 4-5-4-6-4. During this process, the IMU records attitude data of the MAV and calculates the attitude changes within the motion intervals. The camera captures images of the calibration board at stationary moments, and by identifying the corner points of the checkerboard calibration board, the pose of the calibration board relative to the camera is determined using the PnP algorithm. The host system receives the IMU’s attitude data to identify the motion-stationary critical points of the calibration process, distinguishing the motion intervals. As shown in Figure 8, $R_{\mathrm{cij}}$ and $R_{\mathrm{gij}}$ represent the changes in attitude of the camera and IMU during a single motion interval, and $R_{\mathrm{cw}}$ represents the fixed relative pose between the camera and IMU. Finally, by integrating the attitude changes in the camera and IMU over multiple motion intervals, the extrinsic parameters between the camera and IMU are calibrated using the hand-eye calibration algorithm.

### 3.4. Autonomous Landing Module for MAV

**Figure 9:**
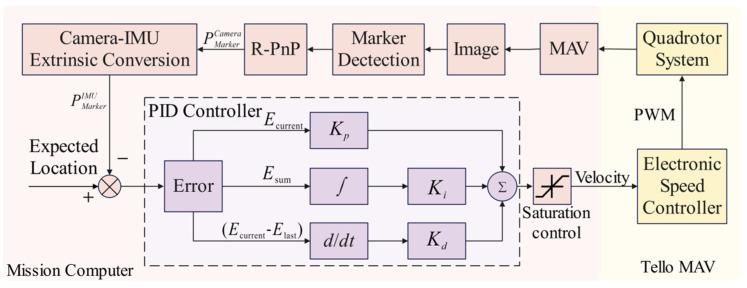
The PID control process for a MAV.

The vision-based autonomous landing module consists of four stages: takeoff, approach, descent, and landing. When the MAV receives the takeoff command, the camera is activated. During this stage, the operator manually controls the MAV, flying it to a distance where the landing area marker is visible. Once the vision system captures the landing area marker, it begins providing feedback on the position, entering a standby state, and readying to receive commands for autonomous landing. After issuing the autonomous landing command, the MAV takes over flight control and enters the approach stage. During this phase, the forward-facing camera captures the landing marker. As the MAV gradually approaches the marker, the camera transitions from using the forward view to the top view to track the marker and obtain the relative position of the marker to the vehicle. As shown in Figure 9, this positional information is input into the PID controller, which then generates velocity control commands for the quadrotor control system. When the MAV reaches the specified threshold distance from the landing marker, it enters the descent stage, gradually reducing altitude and adjusting its position to keep the landing marker in the center of the field-of-view. Upon reaching the preset landing altitude, the MAV hovers. Once stable, it collects the current position data of the landing marker relative to the vehicle. Using these data, displacement commands are issued to the MAV to position it directly above the center of the landing marker. Once the vehicle stabilizes again, the onboard system sends a vertical landing command, ultimately landing the MAV on the designated marker.

The inputs of the PID controller consist of the desired position $E_{\mathrm{expected}}$ and the current position $E_{\mathrm{current}}$. In the MAV autonomous landing task, the value of the desired position is set to 0. Thus, the error value e can be defined as:

(11) $$e=E_{\mathrm{expected}}-E_{\mathrm{current}}=-E_{\mathrm{current}}$$

The PID controller consists of proportional, integral, and derivative units. Its characteristics can be determined by adjusting the gain coefficients $K_{p}$, $K_{i}$, and $K_{d}$ of these three units. The PID algorithm can be expressed by the following formula:

(12) $$\begin{array}{ll} u\left(t\right) & =K_{p}e\left(t\right)+K_{i}\int_{0}^{t}e\left(\tau \right)d\tau +K_{d}\frac{de\left(t\right)}{dt}\\ & =K_{p}\left[e\left(t\right)+\frac{1}{T_{i}}\int_{0}^{t}e\left(\tau \right)d\tau +T_{d}\frac{de\left(t\right)}{dt}\right] \end{array}$$

where $T_{i}$ is the integral time constant, $T_{d}$ is the derivative time constant, t is the current time, and τ is the integral variable, with values ranging from 0 to the current time t. In the equation, $u\left(t\right)$ represents the control output of the controller at time t. In practical implementation, since flight control is a type of sampled control, the control output can only be calculated based on the deviation at the sampling times, unlike continuous output in analog control. Therefore, the flight control of the MAV uses a discrete version of the position-based PID control algorithm. Assuming the sampling time is T, the integral term in Equation (12) can be approximated by the following formula:

(13) $$\int_{0}^{t}e\left(\tau \right)d\tau =T\sum_{j=0}^{k}e_{j}$$

The derivative term of the PID can be approximated as:

(14) $$\frac{de\left(t\right)}{dt}=\frac{e_{k}-e_{k-1}}{T}$$

Thus, the discrete position-based PID expression can be obtained, and Equation (12) can be simplified to:

(15) $$\begin{array}{ll} u_{k} & =K_{p}\left\{e_{k}+\frac{T}{T_{i}}\sum_{j=0}^{k}e_{j}+\frac{T_{d}}{T}\left[e_{k}-e_{k-1}\right]\right\}\\ & =K_{p}e_{k}+K_{i}\sum_{j=0}^{k}e_{j}+K_{d}\left[e_{k}-e_{k-1}\right] \end{array}$$

When the sampling period is sufficiently small, the above approximations provide results that are accurate enough, and the discrete control process closely resembles the continuous control process. In a position-based PID controller, the error continues to accumulate even when the integral term reaches saturation. Therefore, when $\sum_{j=0}^{k}e_{j}$ reaches the maximum integral limit, the integral action should be stopped. To address this, we set the current deviation e to a flag value δ every ten frames. When the deviation $e_{k}$ in a subsequent frame satisfies the following condition:

(16) $$e_{k}\leq \delta -100$$

Then $\sum_{j=0}^{k}e_{j}$ is initialized to 0. This is carried out to allow the MAV to normally increase its speed during the integral accumulation phase, and to reset the integral amount when saturation occurs in order to control the speed. Finally, the PID output is converted to velocity:

(17) $$\left\{\begin{array}{l} v=35,u_{k}\geq 350u_{k}\\ v=P*0.1,u_{k}<350 \end{array}\right.$$

The velocity v will be output to the MAV’s electronic speed control unit, and through the digital output of the microprocessor, it will control the quadrotor’s circuit system.

## 4. Experiments

To validate the effectiveness of the method proposed in this paper, three experiments were conducted, including landing marker detection, indoor autonomous landing experiments, and outdoor autonomous landing experiments. The landing marker detection experiment was used to evaluate the performance of the coarse-to-fine pose estimation algorithm presented in Section 3.1. The indoor autonomous landing experiment was conducted to assess the landing performance of the proposed method in a stable indoor environment for the MAV, while the outdoor autonomous landing experiment was aimed at evaluating the landing performance of the method in an outdoor environment with the wind.

### 4.1. Landing Marker Detection Experiment

**Figure 10:**
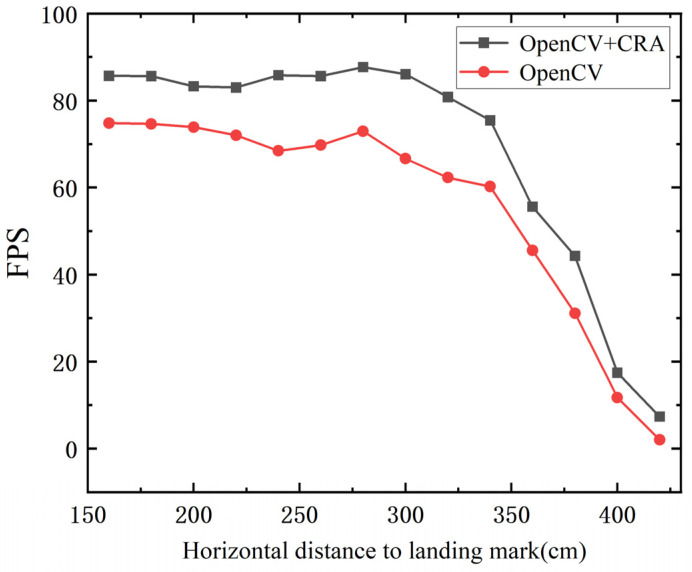
The average frame rate of landing marker recognition.

The coarse-to-fine MAV pose estimation algorithm mainly consists of two parts: landing marker recognition and pose estimation. Since the pose estimation algorithm transforms input image point coordinates into an output extrinsic matrix, its computation speed is relatively stable. However, the performance of landing marker recognition is influenced by factors such as the size of the marker, flight altitude, and horizontal distance, resulting in variations in recognition performance. Therefore, the recognition performance of the landing marker is crucial. To verify the performance of the fusion between the OpenCV ArUco marker recognition algorithm and the Contour Recognition Algorithm (CRA), the MAV was fixed on a tripod at a height of 150 cm, and an ArUco landing marker with an outer size of 25 cm × 25 cm was placed horizontally on the ground. The MAV was positioned horizontally at distances ranging from 160 cm to 420 cm from the center of the landing marker, with measurements taken every 20 cm. The average frame rate of successful recognition over 30 s was monitored as a performance evaluation metric. As shown in Table 1 and Figure 10, the fusion of the OpenCV ArUco marker recognition algorithm and the Contour Recognition Algorithm (CRA) resulted in a noticeable improvement in recognition rate compared to using the OpenCV ArUco marker recognition algorithm alone, with an average frame rate increase of 12.55 FPS.

This improvement is attributed to the CRA, which enhances the tolerance in extracting the contours of ArUco markers, increasing the likelihood of detecting the ArUco marker in the early stages of the algorithm. By incorporating more targeted filtering criteria, it can accurately select the ArUco marker contour from numerous contours. Considering the camera’s sampling rate of 30 FPS, if the landing marker recognition frequency exceeds the camera’s sampling rate, real-time detection can be achieved. As shown in Table 1, within the horizontal distance range of 160 cm to 380 cm, the frame rate of landing marker recognition exceeds 40 FPS. However, in the range of 400 cm to 420 cm, limited by the resolution and depth of field of the camera, the position of the landing mark is beyond the visible distance of the lens, and the image becomes blurred. At the same time, due to the limited shooting angle, the angle between the landing marker and the camera’s optical axis becomes smaller, and the width of the image becomes narrower, making it difficult for algorithms to recognize the external outline of the marker. And, thus, the recognition frame rate drops significantly. Even at a distance of 420 cm, the fusion method of OpenCV and CRA still outperforms using OpenCV alone, greatly improving the MAV’s success rate in capturing markers at long distances, thus accelerating the transition from manual control to automatic guidance mode.

### 4.2. Indoor Autonomous Landing Experiment

The indoor experimental site is setup in the office of daily work, with an area of 40 square meters. The lighting condition is indoor LED panel lighting, and the use of curtains blocks external light interference. There are many desks and experimental platforms in the site, so the flight path of the MAV is usually set in the aisle.

**Figure 11:**
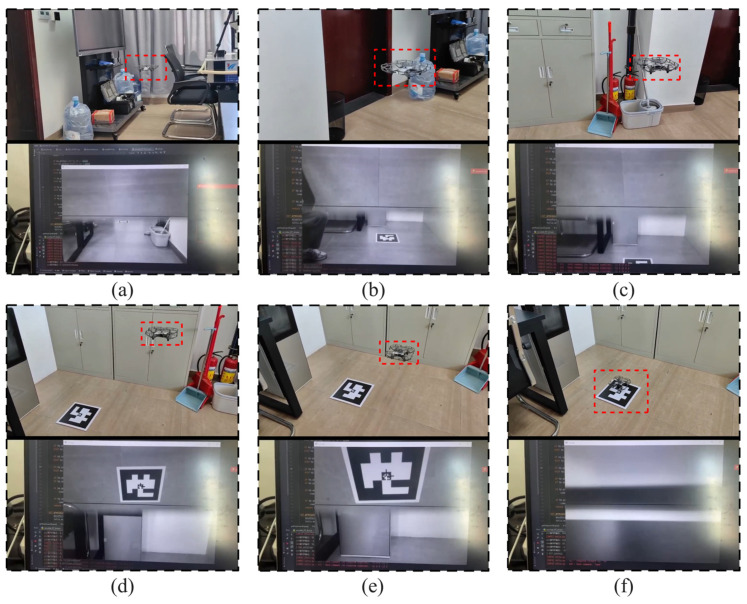
The process of the MAV’s indoor autonomous landing experiment: (**a**) the landing marker is captured in the forward view; (**b**) flying towards the landing marker; (**c**) gap in landing marker capture; (**d**) the marker is captured in the top view; (**e**) descending to the designated altitude; (**f**) displacing above the marker and then vertically landing.

The MAV is placed at a location far from the landing marker for takeoff. It is manually flown to an area where the landing marker can be captured in the forward view, issue an autonomous tracking command to the MAV immediately, after which it enters the autonomous landing phase. As shown in Figure 11, images (a) to (f) illustrate the complete process of the MAV’s autonomous landing.

**Figure 12:**
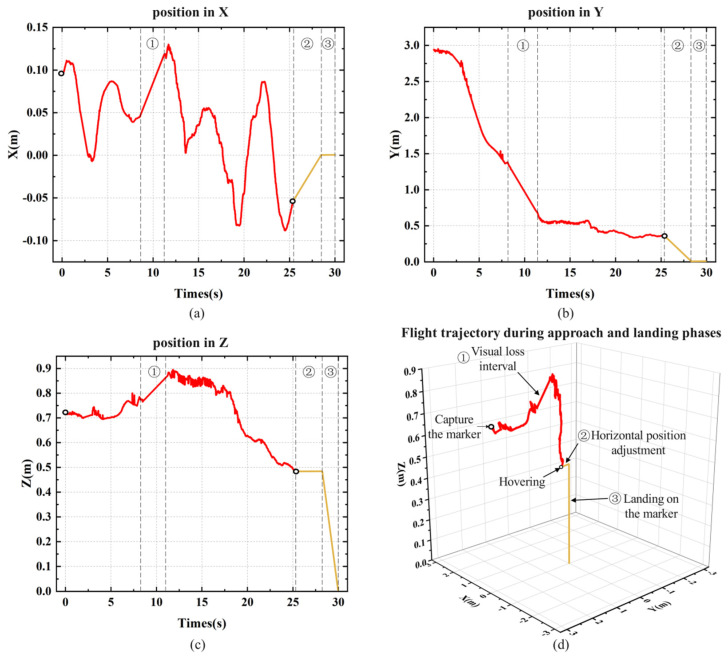
The flight trajectory of the MAV’s indoor autonomous landing experiment: (**a**) *X*-axis position; (**b**) *Y*-axis position; (**c**) *Z*-axis position; (**d**) three-axis flight trajectory during the approach and landing phases.

During the experiment, the X, Y, and Z axis coordinates of the landing marker’s center relative to the MAV’s coordinate system were recorded. Starting from the moment the MAV began autonomous landing, the time corresponding to the coordinate information of each frame was also logged. Figure 12 shows the position visualization curve from one of the landing experiments. Section 1 represents the visual loss interval, which occurs when the landing marker is in the blind spot between the forward and top views during the MAV’s approach, as shown in Figure 11c. In this interval, the MAV temporarily loses its localization and continues to approach the landing marker with the same speed and direction as before the loss, until the marker reappears in the top view. Section 2 represents the horizontal adjustment process before the MAV descends vertically. By calculating the average X and Y axis coordinates during hovering, corresponding displacement commands are sent to the drone. Once the MAV is horizontally aligned above the center of the landing marker, it enters Section 3 for the vertical landing process.

During the approaching stage, the MAV is affected by indoor airflow, causing small lateral deviations in the body. Figure 12a shows that when the MAV deviates from the landing marker along the *X*-axis, the PID controller adjusts the MAV’s displacement, keeping the deviation within ±15 cm. Figure 12b shows the MAV’s displacement along the *Y*-axis, where the flight speed gradually decreases as the MAV becomes closer to the marker, eventually stabilizing within the threshold range to hover. The purpose of hovering is to stably collect the position of the fuselage relative to the landing marker. After collecting position data, the MAV will perform horizontal displacement based on the *x*-axis and *y*-axis positions, displacing it directly above the landing marker, and finally performing vertical landing. Figure 12c records the absolute change in the height of the landing marker relative to the MAV’s coordinate system during the landing process.

**Figure 13:**
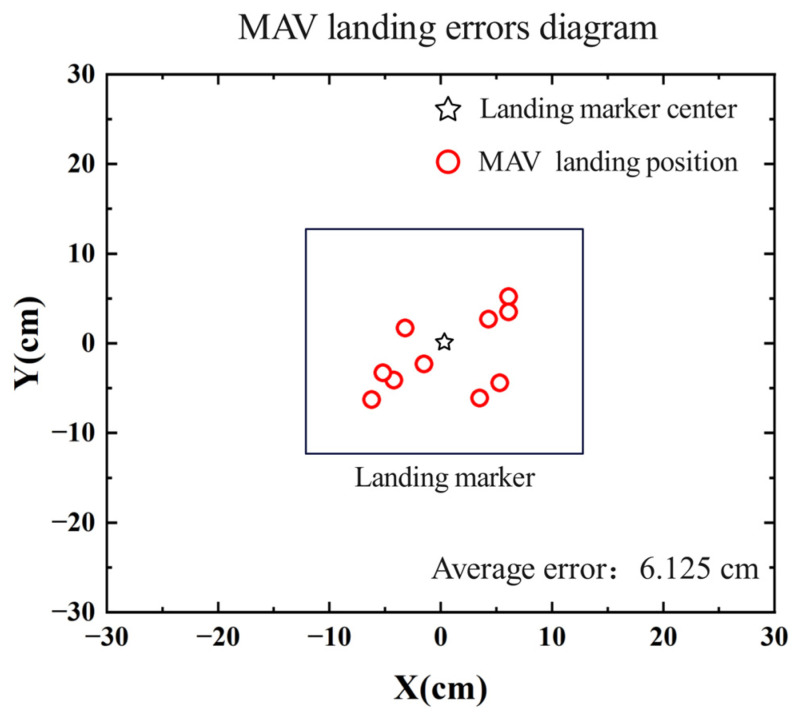
Diagram of MAV autonomous landing error.

To verify the landing accuracy of the MAV, 10 consecutive autonomous landing experiments were conducted. In each experiment, the MAV took off from a different position, and after manually controlling it to capture the landing marker in the forward view, it proceeded to land autonomously. The position of the landing marker center relative to the MAV’s body after landing and the time consumed for each step during the flight was recorded. The distribution of the MAV’s landing positions in the experiments is shown in Figure 13. Table 2 presents the time statistics for each stage of the MAV’s landing process, where Step 1 represents the process from capturing the landing marker in the forward view to capturing it in the top view. Step 2 represents the process from capturing the landing marker in the top view to the MAV descending to a certain altitude and hovering. Step 3 represents the horizontal displacement adjustment process, and Step 4 represents the vertical landing process.

As shown in Figure 13, in the ten consecutive landing experiments, the MAV successfully landed on the landing marker each time, with an average error of 6.125 cm, indicating a certain level of stability in the autonomous landing method. In Table 1, the average time consumed across the 10 experiments is 33.52 s, with Steps 1 and 2 accounting for the largest portions of the time. The maximum time consumed for Step 1 is 20.71 s, with an average of 16.42 s, while Step 2 has a maximum of 14.61 s and an average of 11.56 s. The main reason for the time variance is that when the MAV camera first captures the landing marker, the marker is often off-center in the camera’s view. This results in the MAV needing to adjust its *X*-axis position during the approach or descent. The greater the initial offset, the more time is required to correct the position during the approach or descent. The time consumed in Steps 3 and 4 is relatively similar.

To analyze the level of the experimental results, we compared several works that recorded experimental data, as shown in Table 3. These works also completed the task of autonomous drone landing. Among them, Ref. [24] used a dual-camera setup with forward-facing and downward-facing cameras; Ref. [25] also used a DJI Tello MAV with a reflective lens installed; Ref. [28] employed larger markers for autonomous landing; and Ref. [29] used a GPS-based landing method.

From the comparison results, it can be seen that Ref. [24] and the method proposed in this paper both feature dual perspectives with forward and top views. However, Ref. [24] relies on a dual-camera system, whereas the method in this paper extends the dual view using a single-camera system, making it less demanding in terms of equipment. Ref. [25] and our method show higher landing accuracy, but the field-of-view in our method is larger than that of Ref. [25], and the maximum horizontal distance for positioning is twice as much, giving our method an advantage in long-distance positioning. In terms of maximum horizontal positioning distance, Refs. [28,29] achieve the highest results. Ref. [28] benefits from using larger markers, while Ref. [29] leverages the remote positioning capabilities of GPS. However, GPS-based methods have a positioning accuracy of only 1–3 m, which is far less precise than vision-based methods.

### 4.3. Outdoor Autonomous Landing Experiment

**Figure 14:**
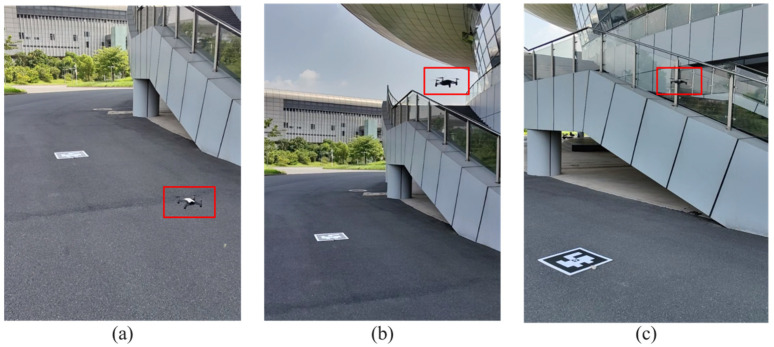

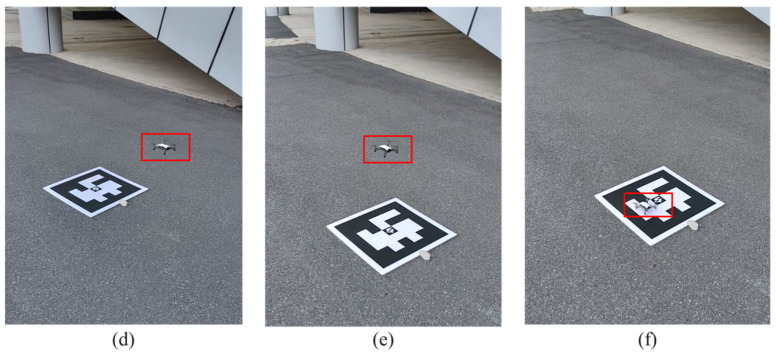
The process of the MAV’s outdoor autonomous landing experiment: (**a**) takeoff process; (**b**) hovering after increasing flight altitude; (**c**) autonomous landing approach process; (**d**) descending process; (**e**) move to the position above the marker; (**f**) vertical landing.

To verify the applicability of the proposed autonomous landing method in outdoor windy environments, an open-air area was selected as the experimental site, and an ArUco landing marker with outer dimensions of 52.5 cm × 52.5 cm was used. The MAV was placed 5 m away from the landing marker for takeoff, and then the flight altitude was raised to 2 m. When the landing marker was captured in the MAV’s forward view, the autonomous landing command was issued. As shown in Figure 14, it illustrates the MAV’s flight process during the outdoor autonomous landing.

**Figure 15:**
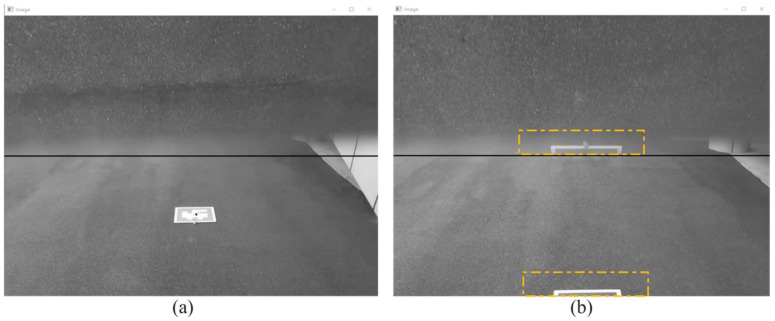

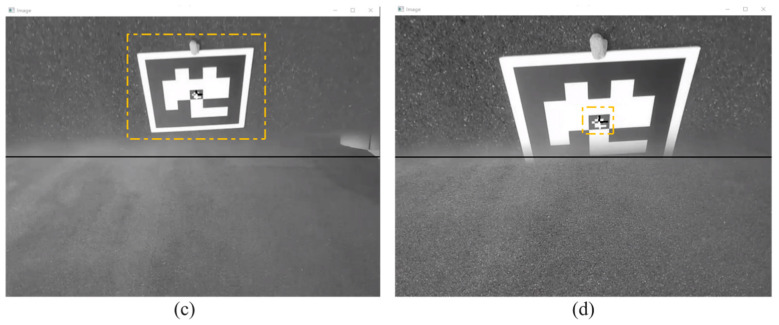
Dual-perspective images of the MAV’s outdoor autonomous landing experiment: (**a**) Forward view capturing the landing marker; (**b**) Blind spot between the forward and top views; (**c**) Top view capturing the outer ArUco marker; (**d**) Top view capturing the inner ArUco marker.

Wind is one of the main factors affecting drone flight in outdoor environments. For the DJI Tello MAV, which weighs only 80 g, autonomous landing in windy conditions is particularly challenging. Compared to indoor experiments, we set a faster *Z*-axis descent speed and shorter landing response time to mitigate the impact of wind on the MAV’s horizontal displacement adjustments, though this comes at the expense of some landing accuracy. Figure 15 shows the camera view of the MAV’s autonomous landing process. In Figure 15b, it is clear that there is a positioning blind spot between the two perspectives during the MAV’s approach to the marker. Since the flight altitude in the outdoor environment is higher than indoors, this blind spot is smaller. When the marker disappears from the forward view, it subsequently appears in the top view. This makes the blind spot between the views almost negligible for autonomous landing. When the marker is fully displayed in the view, as shown in Figure 15c, the camera will recognize the outer ArUco marker. However, if the MAV’s descent is affected by wind or the size of the top view limits the visibility of the outer ArUco marker, the camera will recognize the inner ArUco marker instead. Since the inner ArUco marker measures only 7.5 cm × 7.5 cm, which is much smaller than the outer marker, it can still be recognized even if there is a momentary deviation in the flight attitude, allowing for correction and maintaining the marker at the center of the view.

**Figure 16:**
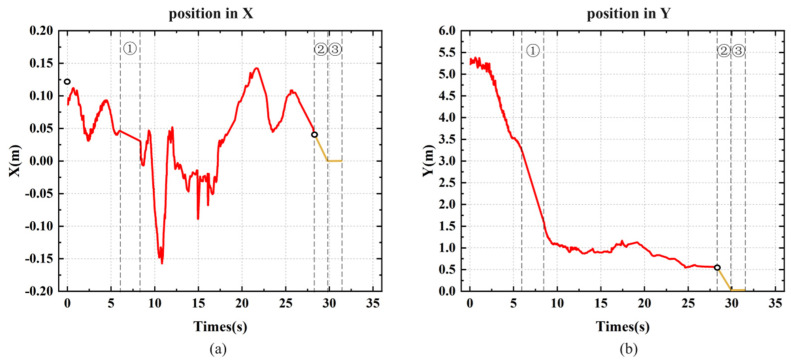

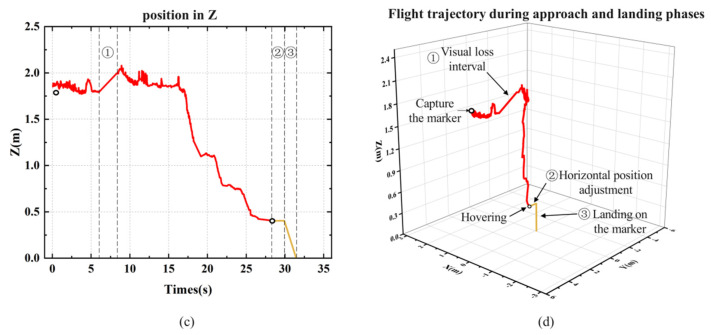
Flight trajectory of the MAV’s outdoor autonomous landing experiment: (**a**) *X*-axis position; (**b**) *Y*-axis position; (**c**) *Z*-axis position; (**d**) Three-axis flight trajectory during the approach and landing phases.

Similar to the indoor experiment, as shown in Figure 15, we recorded the MAV’s flight trajectory in the outdoor environment. In Figure 15a, it is evident that the maximum deviation along the *X*-axis exceeded 15 cm, which is larger than in the indoor experiment, and the *X*-axis curve is less smooth compared to the indoor experiment. This reflects the effect of crosswinds on the MAV’s *X*-axis position during flight in the outdoor environment. Due to the faster *Z*-axis descent speed and shorter landing response time, as seen in Figure 16c, the slope of the *Z*-axis curve during descent is steeper compared to the indoor environment. Despite the starting position being farther and the flight altitude being higher than in the indoor experiment, the landing time was only about 2 s longer than in the indoor test.

From the results of both indoor and outdoor experiments, it is evident that the mirror-based field-of-view extension system successfully combines the advantages of forward-facing positioning and downward-facing landing. This provides the possibility for autonomous landing for MAVs equipped with only a forward-facing camera. If relying solely on the original camera for positioning, the MAV would lose its positioning during the approach, making it impossible to complete the landing.

## 5. Conclusions

This paper presents an MAV autonomous landing technology based on a mirror field-of-view extension system and a vision-based autonomous landing method. The forward-facing camera of the MAV employs a reflective lens to extend its field-of-view, expanding the top view while retaining the original forward view. This allows the MAV to capture the landing marker from varying distances during the autonomous landing process. Additionally, a camera-IMU extrinsic calibration method is introduced to determine the pose relationship between the MAV’s body and the landing marker, obtaining positional data of the landing marker relative to the MAV’s body. Indoor and outdoor flight experiments were conducted using a DJI Tello MAV to validate the proposed autonomous landing method. The indoor landing accuracy achieved 0.06 m, with an average landing time of 33.52 s. The results demonstrate that this strategy enables an MAV equipped only with a forward-facing camera to autonomously complete landing tasks in both indoor and outdoor environments, highlighting the application potential of this type of MAV. Future work will focus on developing a more optimized PID controller to further enhance the speed of MAV autonomous landings.

## Figures and Tables

**Table 1:** The average frame rate of landing marker recognition.

	160 cm	180 cm	200 cm	220 cm	240 cm	260 cm	280 cm
OpenCV + CRA (FPS)	85.65	85.59	83.24	82.96	85.80	85.60	86.02
OpenCV (FPS)	74.82	74.63	73.88	72.03	68.44	69.74	72.94
	300 cm	320 cm	340 cm	360 cm	380 cm	400 cm	420 cm
OpenCV + CRA (FPS)	86.02	80.78	75.44	55.60	44.29	17.43	7.36
OpenCV (FPS)	66.62	62.29	60.21	45.56	31.12	11.76	2.02

**Table 2:** Time consumed for each step in the MAV’s autonomous landing process.

	STEP 1	STEP 2	STEP 3	STEP 4	Total
1	15.59	8.89	2.01	3.02	29.51
2	20.71	13.20	2.31	3.21	39.43
3	16.20	12.98	2.51	3.21	34.90
4	16.64	11.53	2.21	3.32	33.70
5	17.20	6.73	2.11	3.52	29.56
6	14.85	12.21	2.11	2.91	32.08
7	12.84	10.30	2.31	3.82	29.27
8	17.01	13.30	2.12	3.22	35.65
9	18.86	14.61	2.41	3.42	39.30
10	14.33	11.84	2.41	3.22	31.80
Average	16.42	11.56	2.25	3.29	33.52
Units: s					

**Table 3:** Comparison of landing strategies.

Methods	Ref. [24]	Ref. [25]	Ref. [28]	Ref. [29]	Ours
field-of-view	Forward & Top	Top	Top	None	Forward & Top
Accuracy [m]	0.16	0.05	0.15	1–3	0.06
Distance [m]	1.2	2	20	20	4
Marker Size [cm]	r = 13.5	22.5 × 17.5	80 × 80	None	25 × 25
Vision Resolution	1280 × 720	1280 × 720	None	None	960 × 720
Outdoor	No	Yes	Yes	Yes	Yes

## Data Availability

The original data presented in the study are openly available in [GitHub] at [https://github.com/FSlxf/micro-UAV-landing-strategy] (accessed on 18 October 2024).
